# Supplementary material for: NAC Transcription Factor GmNAC035 Exerts a Positive Regulatory Role in Enhancing Salt Stress Tolerance in Plants
Source: Plants (Basel). 2025 May 5;14(9):1391. doi: 10.3390/plants14091391 (PMC12073727; doi:10.3390/plants14091391)
Supplement: Supplementary file 1 [file plants-14-01391-s001.zip › Supplemental Figure.pdf]

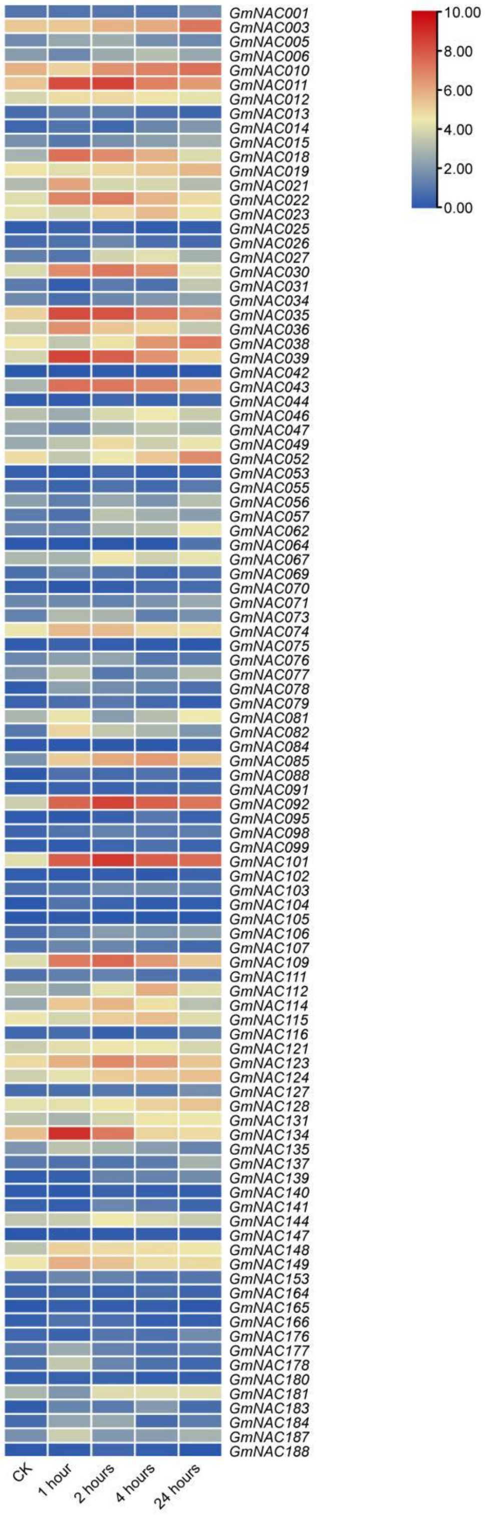


Figure S1: Expression profiles of *GmNAC* transcription factors in soybean in response to salt stress. Under the stress condition of 100 mM NaCl, the expression of genes was induced by salt stress at least at one time-point, with the criterion of a fold - change in expression greater than 2. The RPKM normalized values of genes were log$^{2}$-transformed and visualized as a heatmap. The gene IDs are shown in Supplementary Table S1.

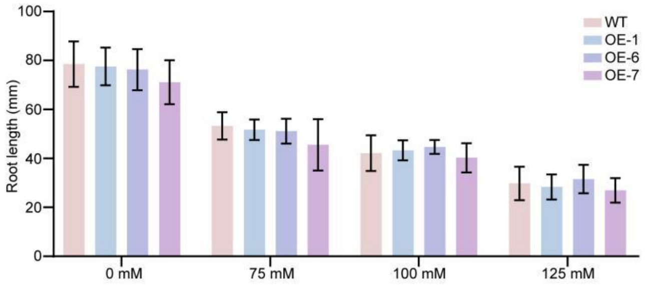


Figure S2: Statistical analysis of root lengths of wild-type Arabidopsis and overexpression lines under salt stress at different concentrations. *n* = 20. The data represent the mean ± SD.

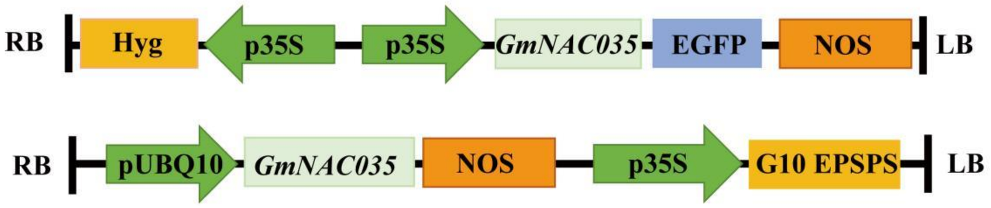


Figure S3: Schematic diagram of the vector used in this study. The structure of the pCAMBIA1305-GFP used for subcellular localization and the pCAMBIA 1300 vector used for Arabidopsis thaliana genetic transformation
